# Balance perturbation system to improve balance compensatory responses during walking in old persons

**DOI:** 10.1186/1743-0003-7-32

**Published:** 2010-07-15

**Authors:** Amir Shapiro, Itshak Melzer

**Affiliations:** 1 Department of Mechanical Engineering, Faculty of Engineering, Ben-Gurion University of the Negev, Beer-Sheva, Israel; 2 Department of Physical Therapy, Faculty of Health Sciences, Ben-Gurion University of the Negev, Beer-Sheva, Israel

## Abstract

Ageing commonly disrupts the balance control and compensatory postural responses that contribute to maintaining balance and preventing falls during perturbation of posture. This can lead to increased risk of falling in old adults (65 years old and over). Therefore, improving compensatory postural responses during walking is one of the goals in fall prevention programs. Training is often used to achieve this goal. Most fall prevention programs are usually directed towards improving voluntary postural control. Since compensatory postural responses triggered by a slip or a trip are not under direct volitional control these exercises are less expected to improve compensatory postural responses due to lack of training specificity. Thus, there is a need to investigate the use balance perturbations during walking to train more effectively compensatory postural reactions during walking.

This paper describes the Balance Measure & Perturbation System (BaMPer System) a system that provides small, controlled and unpredictable perturbations during treadmill walking providing valuable perturbation, which allows training compensatory postural responses during walking which thus hypothesize to improve compensatory postural responses in older adults.

## Introduction

Postural control is the foundation of our ability to move independently. Acute injuries, including traumatic brain and spinal cord injuries, Hip fracture and even death occurring as a result of falls in old adults [1]. In the older adults about one out of three individuals fall at least once a year [2]. Falls are the leading cause of accidental death in the elderly population [3]. The cost has been estimated to be nearly $10 billion for one year [4-6]. Consequently, there is a need to develop new technologies that will improve interventions for reducing falls and increasing quality of life in older adults.

The benefits of exercise with respect to general health, strength, and balance have been long documented in the physical exercise literature [7-16]. However, research studies investigating exercise as a means of falls prevention in older adults have shown controversial results. Several studies show that exercise prevents falls [17-22] and other studies have shown no reduction in falls [23-25]. The controversial results may be the result of the flaw in many balance training programs ignoring a basic principle of physical training, the concept of specificity. The majority of falls occurs during walking [26] and results from unexpected perturbations. In spite of this, most balance training regimens only include voluntarily controlled exercises [14-25], that do not include perturbation exercises to improve compensatory postural responses during walking, which may improve the ability to prevent falling when a person loses his/her balance.

The postural responses triggered by a slip or a trip are not under direct voluntary control [27-29]. These postural "reflexes", initiated by external postural perturbations, lead to activation of specific recovery strategies. These recovery strategies are not under volitional control and thus the optimal means for training compensatory responses will involve unexpected external perturbation exercises during walking. The Balance Measure and Perturbation System (BaMPer System) described here triggers postural "reflexes" to improve balance responses is designed to supply the patient with an unexpected acceleration during treadmill walking.

Wolfson et al. [30] were able to demonstrate improvements in balance function in old adults using intensive balance training that included equilibrium control exercises of firm and foam surfaces and/or weight training followed by 6 months of low intensity Tai Chi training. Oddsson et al. [31] proposed a specific training program that involves use of unpredictable, multi-directional perturbations to evoke stepping responses in elderly persons. Mansfield et al. [32] used of a perturbation platform that moves suddenly and unpredictably during standing on the platform in one of four directions as part of a balance training program. Rogers et al. [33] showed that either voluntary or waist-pull-induced step training reduced step initiation time. The above-mentioned studies [30,32,33] and perturbation systems previously used in research, train compensatory responses during up-right standing and not during walking, this is not the optimal means for training compensatory responses during walking since it lacks the specificity principle of exercise physiology.

Miziaszek and Krauss [34] used forwards and backwards perturbations while walking on a motorized treadmill. These were perturbations of center of mass that were randomly applied at the pelvis compared with the base of support perturbations that is applied by the BaMPer System suggested here, both type of perturbation are relevant to 'real-life' postural perturbations and responses. Shimada et al. [35] used bilateral separated treadmill whereas each of the separated belts where run in a different speed to perturb normal gait. Bhatt and Pai, [36] exposed elderly subjects to a slip backward balance loss as a training to improve stepping reactions. The unidirectional slip (backwards only) is the major drawback of the system, since the direction of perturbation was expected after several exercises. Thus it seems that this is not the optimal means for training compensatory responses to different directions.

## System Description

The basic requirements for the BaMPer system are based on Oddson et al. results [37]. Oddson et al. applied perturbation of which the maximal acceleration is 9.81 m/sec^2 and the maximal velocity is 0.7 m/sec. Therefore while designing the BaMPer system we chose the system to be able to apply maximal acceleration of 9.81 m/sec^2, and to reach maximal velocity of 0.8 m/sec. The maximal displacement during perturbation was chosen to be 10 cm to any direction in the horizontal plane in order to simulate bumping into a small obstacle.

**Figure 1:**
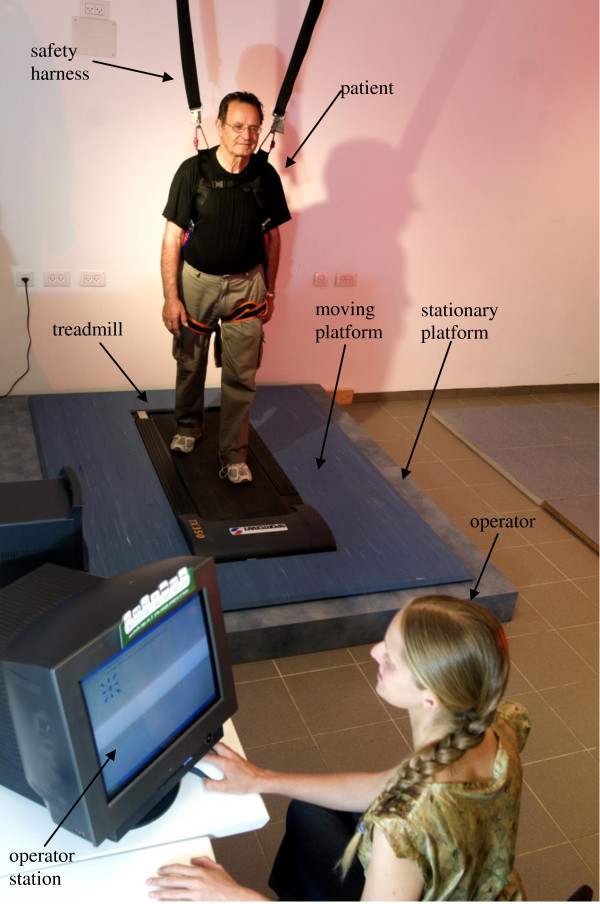
**Photo of the the BaMPer system**. The system is compose of a motor-driven treadmill, mounted on a moving platform, motion controller, safety harness and an operator station (see the text for more details).

The system is composed of a motor-driven treadmill (weigh 45 lbs), 140 cm length and 60 cm wide, mounted on a moving platform, motion controller, and an operator station (Figure 1). No person weighing over 250 pounds should use the treadmill. The dimensions of the moving platform are 160 cm wide and 200 cm long. The moving platform is mounted on linear slides, which allow it to translate in any direction in the plane. Two linear actuators are responsible for moving the platform longitudinally, laterally, or any combination of those directions. The motion controller controls the motion of the two motors such that the motion is along the trapezoidal velocity profile (i.e., accelerating, moving at a constant velocity, decelerating). The operator's station serves as the user interface of the system and provides the therapist with the ability to control all training parameters including maximal acceleration, number of repetitions, and time intervals. The computer also saves a log file of the training protocol for future use. The entire perturbation system weighs about 130 kg. The perturbation system maximum power consumption is 3.6 kW not including the treadmill consumption. And the building cost of the prototype was about $17,000. The following describe the three main components of the system: hardware, motion control, software design and user interface, and finally we discuss some the safety issues.

### A. Hardware

**Figure 2:**
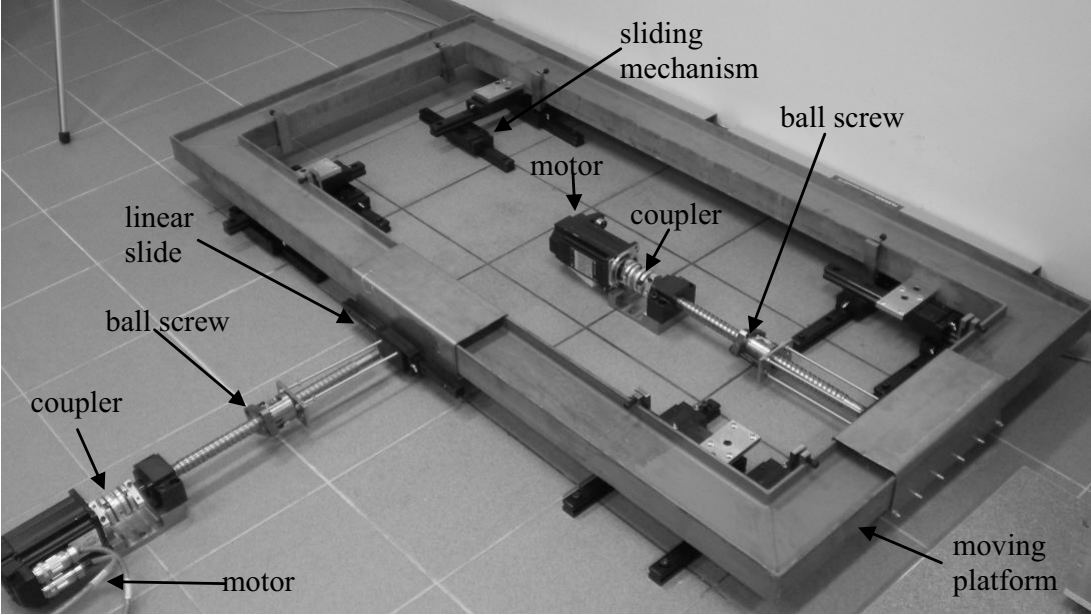
**Photo of the uncovered BaMPer system**. The moving platform, linear slides, and two linear actuators which are responsible for moving the platform longitudinally, laterally, or any combination of those directions.

The hardware of the system (table 1) includes the following components: treadmill, moving platform, linear slides, linear actuators, and ball rollers. The uncovered BaMPer system with the treadmill removed is shown in Figure 2.

**Table 1:** List of system's components and their model numbers.

Component	Manufacturer and model number
Main linear slides	ABBA BRH30BL
Drive unit: AC servomotor	Rockwell Automation MPL-A330P-HJ22AA
Flexible coupling	Huco flexible coupling p/n 670.52.42.40
Ball drive unit	Kuroda GG2510DS-BALR-0533C-C5S
Supporting bearing unit	Kuroda BUK20A
linear slide between the nut and the moving frame	ABBA BRH25BL
Motion Controller	ACS SpiiPlus CM-2-BE-MO

The moving platform is mounted on four sliding mechanisms to allow motion in both longitudinal and lateral directions. Each of the four sliding mechanisms is composed of three main linear slides mounted in an H-like shape. Each of the two driving units is composed of an AC servo motor connected through a coupler to a ball screw. The nut of the ball screw is connected through a linear slide to the moving frame. The reason for the additional linear slide between the nut and the frame is that the frame can be moved perpendicularly by the other drive unit. For the drive unit, we used AC servo motors with 1800 W power, maximal speed of 5000 rpm, and peak torque of 11.1 Nm. A flexible coupler transfers the required motion from the motor to the ball drive unit. Position sensing is accomplished by optical encoders mounted on the back side of each motor. Limit switches are mounted on the base stationary part of the system ate the maximal travel distance.

### B. Motion Control

**Figure 3:**
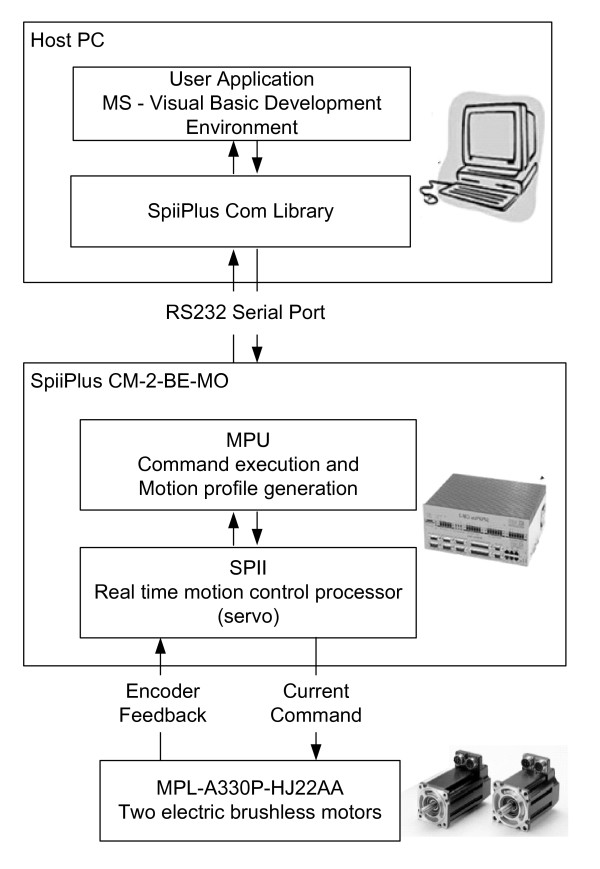
**Motion control diagram**.

The motion control system is based on the ACS SPiiPlus-CM controller. In our system the host PC serves as a user interface and as a high level programming environment. The control architecture is described in Figure 3.

**Figure 4:**
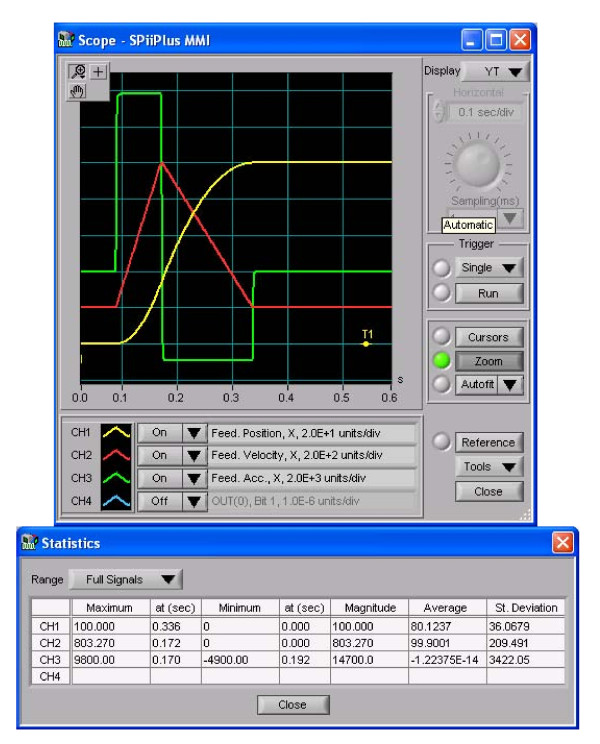
**Motion parameters during experiment**. Graphs of the position (mm), velocity (mm/sec), and acceleration (mm/sec^2^) during perturbation experiments are shown. Note those are actual measurements taken during perturbation experiment.

The control program, which will be described hereafter, uses the SpiiPlus Com Library to communicate with the two-axis motion controller and brushless motor drivers. Communication between the PC and the controller is simple RS232 serial communication. The controller receives from the PC program the required motion parameters, which are the target position, maximal velocity, acceleration, and deceleration. The controller has an internal motion profile generator that generates a trapezoidal velocity profile. In our case, where acceleration is the important parameter, we use a triangular velocity profile where the platform accelerates in order to generate the required perturbation, and then decelerates to zero velocity. The controller has a real time CPU that controls the motion using PID control law. The internal driver sends current commands to the motors, and the controller receives position feedback from optical encoders mounted on the back of each motor. Graphs of the position, velocity, and acceleration during perturbation experiments are shown in Figure 4.

### C. Software Design and User Interface

The program that serves as the system's user interface is written in Microsoft Visual Basic 2008 and runs on the host PC. The application is a Windows form application and contains four tabs: communication, setting parameters, testing, and run experiment.

**Communication tab**: The communication tab allows opening and closing the communication port to the ACS controller. It also reminds the operator to check if the safety harness is secured. In addition, it automatically calibrates the travel range of each of the motors and moves the platform into the home position at the center of the working range. This calibration is done by slowly moving the platform until it reaches the limit switches at the maximal travel distance and then setting the position measured by the motors' encoders to be accurately the actual position.

**Set Parameters tab**: this tab enables changing the minimal and maximal values of the motion profile parameters. It also enables setting the number of perturbations during a single experiment or training series, and the time delay between two consecutive perturbations. For each perturbation to be executed the system will randomly select each parameter within the range specified by the minimal and maximal values.

**Testing tab**: This tab allows applying a single perturbation in a manually selected direction.

**Run Experiment tab**: This tab is the most important one, since from here the operators actually starts the training sequence in which a series of perturbations will be applied to the patient. The tab presents several items, first are the start and stop buttons for starting the training or stopping it. Then there is the number of current perturbations within the series (initial value is zero), and the total time left for the current run. The operator can provide a filename for a log file that contains the run parameters. On the right there is a box that will contain a graph of the platform velocity during the perturbation interval. On the bottom there is a table containing all the motion parameters that have been randomly selected for the perturbation executed.

### D. Safety

Safety is an extremely important issue since we apply perturbation to an older patient walking and that may cause him or her to fall. During the training the treadmill will continue to run also after platform motion (e.g. perturbation of balance), even though one foot is located on the surrounding surface outside the treadmill. The subject will be instructed to recover from loss of balance due to perturbation by stepping outside the treadmill and than return to walk on the treadmill as fast as he possibly can, which is the most important part of the training regimen. Results of a pilot study show that during lateral perturbations young individuals respond by quick stepping response off the platform to the opposite direction of the perturbation and recovered by stepping back quickly into the treadmill. In anterior posterior platform perturbations young individuals responded by a quick increase (in backward perturbations) or quick decrease (in forward perturbations) of walking speed. Low accelerations did not evoked stepping response however quick movement of the upper body to the opposite side of the perturbation seen to recover movement of the bodies' center of mass. In case the subject fail to recover and falls, safety cables that connect the subject waist to the treadmills control panel will stop the treadmills from its continuous motion. Furthermore, to prevent any injury during loss of balance and fall initiation, the patient is wearing a safety harness that will arrest the fall before the patient's knees touch the ground. Examples of such a safety harness are the Skylotec G-0904 or the PN12 harness. The safety harness is hung from the ceiling by two ropes above the patients. However, for stability reasons the ropes do not hang straight from the ceiling, but in a diagonal such that the distance between the connection points of the two ropes on the ceiling is about 2 m. When the rope is hanged in diagonal it is capable to apply much larger horizontal force in order to keep and stabilize the patient at the center. The treadmill works as an ordinary treadmill and only the therapist controls the speed/stops the treadmill and controls the perturbation displacements/velocity/accelerations ranges. If the subject is unable to 'keep up' with the speed a modifications will be made by the therapists.

## Consent

Written informed consent was obtained from the patients for publication of this case report and accompanying images. A copy of the written consent is available for review by the Editor-in-Chief of this journal.

## Competing interests

The authors declare that they have no competing interests.

## Authors' contributions

IM and AS was involved in planning the BaMPer system as well as drafting of the manuscript and have both given final approval of the current manuscript.
